# How does the carboxyl terminus assist folding and ER export of the serotonin transporter?

**DOI:** 10.1186/2050-6511-13-S1-A55

**Published:** 2012-09-17

**Authors:** Ali El-Kasaby, Florian Koban, Michael Freissmuth, Sonja Sucic

**Affiliations:** 1 Institute of Pharmacology, Center for Physiology and Pharmacology, Medical University Vienna, 1090 Vienna, Austria

## Background

The serotonin transporter (SERT) is exported from the ER by recruiting SEC24C to its C-terminus [1]. The same region also provides a docking site for proteinaceous chaperones (HSP isoforms) that assist in folding [2]. Based on these observations, we postulate sequential exchange of the chaperone(s) for the COPII coat as a mechanism to prevent premature ER export of partially folded SERT.

## Methods

SERT (including a series of double and truncation mutants along its C-terminus) and related transporters were screened for their SEC24 isoform dependence, examined by siRNA-induced SEC24 knock-down in HEK293 cells. The cells were transfected with Sec24 siRNAs and, 48 h later, with transporter plasmids (Lipofectamine, Invitrogen), and the effects were determined by substrate uptake and confocal microscopy. GST-fusion proteins comprising the C-terminus of wild-type and mutant SERTs were used for pull-down experiments carried out with SEC24C and SEC24D. To examine the role of heat shock protein (HSP) 90 in regulating the formation of COPII vesicles, we treated HEK293 cells expressing wild-type SERT and its RI-607,608-AA mutant (putative ER export motif site) with geldanamycin, prior to measuring the effect of HSP90 inhibition on transporter trafficking.

## Results

A lysine residue (K610) residing near the putative ER export motif on SERT (RI-607,608) was replaced by tyrosine (Y), the equivalent residue found in NET and DAT, leading to a relaxed preference for SEC24 isoform recruitment; SERT-K610Y no longer relied solely on SEC24C, but rather SEC24D for ER export. We searched for HSP isoforms that bound within or close to the SEC24-binding site. These experiments revealed a role of HSP90 in relaying SERT to SEC24C.

## Conclusions

We show that the preference for SEC24 isoforms can be altered upon mutation of a single residue on the cargo protein. In SERT, lysine 610 appears to have a role in the interaction with SEC24C. We base our conclusion on the following evidence: (i) GST pull-downs showing an interaction with SEC24D, rather than SEC24C, (ii) siRNA-induced knock down of SEC24 isoforms A–D and (iii) co-expression with dominant negative SEC24C/SEC24D mutants, leading to reduced surface expression of the transporter.
